# Experimental Analysis of a Failed Gamma Nail: A Case Report and Literature Review

**DOI:** 10.3390/healthcare12161578

**Published:** 2024-08-08

**Authors:** Mihai Alexandru Cordunianu, Alina Georgiana Vulcu Cordunianu, Iulian Antoniac, Andrei Luca, Marius Niculescu, Cristian Ovidiu Chiriac, Iuliana Corneschi, Cosmin Ioan Mohor

**Affiliations:** 1 Faculty of Medicine, Titu Maiorescu University, 67A Gheorghe Petrascu, 031593 Bucharest, Romania; 2 Faculty of Material Science and Engineering, University Politehnica of Bucharest, 313 Splaiul Independentei, 060042 Bucharest, Romania; 3 Department of Neurologic Recovery, Central Military Emergency University Hospital Carol Davila, 134 Calea Plevnei, District 1, 010825 Bucharest, Romania; 4 Faculty of Medicine, Lucian Blaga University of Sibiu, 10 Victoriei Boulevard, 550024 Sibiu, Romania

**Keywords:** Gamma Nail, implant failure, elderly trauma, trochanteric fracture, experimental determinations

## Abstract

The Gamma Nail represents one of the most popular and efficient implants for treating proximal femoral fractures. Our paper reports a case of a failed Gamma Nail which was used for the surgical treatment of a 69-year-old woman with a right femoral trochanteric fracture due to a car accident. After the surgical intervention, 6 months later, the patient presented to the hospital reporting pain and limited mobility of the right hip. An X-ray was performed at the level of the pelvis, which highlighted the fracture nonunion and the implant failure. The implant removal and its replacement with a dynamic condylar screw system (DCS) was decided. Because Gamma Nail failures are rare occurrences, the implant was subjected to analyses and experimental determinations to find out the cause. For the implant analyses, a stereomicroscope, an optical microscope, and scanning electron microscopy were used. After the tests were conducted, preparation and processing irregularities as causes of the implant failure were eliminated. Also, the experimental analyses showed that the Gamma Nail did comply with chemical composition and microstructure regulations. Thus, it was concluded that the implant failed due to the mechanical overloading caused by surgical technique errors.

## 1. Introduction

With the increasing of life expectancy, the number of elderly trauma patients is expected to increase [1]. In 1997, a study conducted by Gullberg et al. calculated that femoral fractures would double their number from 1990 to 2025 and then double again until 2050 [2]. Trochanteric fractures are frequent in the adult population, affecting especially the elderly [3]. A study conducted by the Swedish Fracture Register from January 2014 to December 2016 which included non-pathological trochanteric and subtrochanteric femoral fractures identified 10,548 patients with a mean age of 82 ± 11 years, and the majority of patients (69%) were females. Most fractures were caused by a fall at the same level (83%) [4]. This type of fracture comes with an increased risk of the loss of mobility and higher morbidity; for this reason, it is recommended to be treated surgically with no delay, providing the opportunity of a quick recovery [5]. The Gamma Nail was first introduced in 1988; the second generation was introduced in 1997 and the third generation in 2006 [6,7,8]. The Gamma 3 Locking Nail System represents one of the most common implants used in the treatment of proximal femoral fractures in the trochanteric region and for bone deformity correction in cases of malunion and nonunion, which are also rarely associated with complications. Considering these data, it is very important to understand the causes that can lead to implant failure in order to determine if they depend on the manufacturing of the implant, or if they are related to surgical procedure or recovery errors. This paper aims to find out, through analyzing the data, the causes that led to the implant failure of the reported patient, and how this situation can be prevented in the future by appropriately choosing material quality and implant design and also avoiding surgical implantation errors or implant overloading by the patient [9].

## 2. The Gamma Nail

The Gamma Nail is based on intramedullary nailing principles during closed surgical procedures. The Gamma Nail appears to have an advantage over the DePuy Synthes Dynamic Hip System (DHS) in treating unstable fractures, due to it requiring less surgical trauma and maintaining better stability [10]. The nail design is based on Kuntcher’s Y-nail and on locking intramedullary nails [11]. This device is composed of a funnel-shaped intramedullary nail with a slight bend to reflect the proximal femoral anatomy and a large proximal opening which features a sliding mechanism for a large cervico-cephalic lag screw. This combination allows for a 25% to 30% reduction in the bending stress of the implant compared to extramedullary implants [12]. The lag screw is also indicated for rotationally unstable fractures. Inserting the Gamma lag screw compacts the cancellous bone, providing additional anchoring, which is highly valuable in osteoporotic bone. It is also possible to obtain distal locking with the help of one screw, static or dynamic. The Gamma Nail, through its biomechanical stability and functional outcome, appeared as an alternative to screw plates, nail plates, and Ender nailing [13,14].

The closed technique by which they are fixed represents an advantage because it helps retain the fracture hematoma, requires a lower operating time, and results in a lower amount of blood loss [15]. The association of an intramedullary fixation in the femoral shaft with a sliding screw in the femoral head enables a controlled collapse of the fracture site and limitation of excessive shaft medialization in unstable intertrochanteric fractures [16]. Another reason for the nail’s popularity is that it becomes fully weight-bearing earlier than alternatives [17].

## 3. Trochanteric Fractures

A trochanteric fracture describes a fracture involving the greater and/or lesser femoral trochanter. In this region, the fractures can be classified as being intertrochanteric, subtrochanteric, avulsion of the greater trochanter, and avulsion of the lesser trochanter [18].

Intertrochanteric femur fractures are described as extracapsular fractures of the proximal femur that appear in the region comprised of the greater and the lesser trochanter, a region composed of trabecular dense bone. This type of fracture can appear in the young and the elderly both, but is more common in the elderly population with osteoporosis. Around 280,000 fractures occur annually in the United States, with almost half of them being intertrochanteric, and by the year 2040 an increase is expected [19]. Another study supports the fact that over 300,000 people over the age of 65 sustain a hip fracture in the United States [20]. The most utilized fracture classifications regarding this fracture are the Evans classification, which is presented in Table 1, and the Arbeitsgemeinschaft für Osteosynthesefragen (AO) classification [21], which is presented in Table 2, but neither of them is considered to be ideal, although there are some changes in the AO classification that should allow for better inter-observer agreement [22,23].

The Evans classification divides this type of fracture into two groups: 

**Table 1:** Evans classification of trochanteric fractures [24].

	Type 1 Fracture	Type 2 Fracture
Group 1	Undisplaced, stable fractures	Reverse oblique, unstable fractures
Group 2	Displaced, but with stable medial cortical apposition	
Group 3	Displaced, unstable fractures with no medial apposition	

The AO system classifies the intertrochanteric fractures as 31A3, where the fracture line passes between the two trochanters, above the lesser trochanter, and below the crest of the vastus lateralis, with the involvement of both cortices.

The 31A3 fracture is subdivided as follows:

1. 31A3.1—simple oblique fracture;
2. 31A3.3—simple transverse fracture;
3. 31A3.3—wedge of multifragmentary fracture, and this is the most common type [25].

Trochanteric fractures can also be contained in the AO classification of proximal femur fractures, and they are classified as follows:

**Table 2:** AO classification of proximal femur fractures (trochanteric fractures classification) [26].

Type A Fractures		
A1—fractures (simple pertrochanteric fracture)	A2 fractures	A3 (intertrochanteric or reverse oblique fracture)
Comminuted fractures, incompetence of the lateral wall	Comminuted fractures, incompetence of the lateral wall	A3.1—simple oblique fracture
A1.2—two-part fracture		A3.2—simple transverse fracture
A1.3—lateral wall intact		A3.3—wedge fracture or multifragmentary

### Gamma Nails Failure

Orthopedic implant failure describes the situation in which the implant is unable to live up to its expected requirements. This includes any complication that is directly related to the implant such as wear, failure, allergic reaction, and dislocations due to material fatigue [27].

A damaged orthopedic implant in the body can cause problems for the patient and disrupt the therapeutic process [28]. 

The most typical sign of an implant failure is ongoing or increasing discomfort in the affected joint, which may decrease the range of motion. Because of this, the patient’s mobility and quality of life may suffer greatly [29]. Joint instability, brought on by an improper implant, may make it hard for the patient to do things like bear weight or carry out everyday tasks. An inability to repair the implant or return the joint to its normal function may result from the bone damage that occurs when an implant fails. Implant failure also raises the risk of infection, which is difficult to treat and can need further surgery. It may be necessary to remove the failed implant and insert a new one during revision [30].

The most common cause of implant failure is represented by implant infection, which leads to implant breakdown that can be associated with the nonunion of a broken bone [30]. 

The Gamma Nail’s indications are represented by the treatment of pertrochanteric, intertrochanteric, and subtrochanteric fracture types AO/OTA 31-A1, 31-A2, and 31 A-3. Also, other indications include the treatment of pathological fractures, treatment of tumoral resections, and revision procedures. The contraindications are represented by fractures of the femoral head and femoral neck fractures [8].

**Figure 1:**
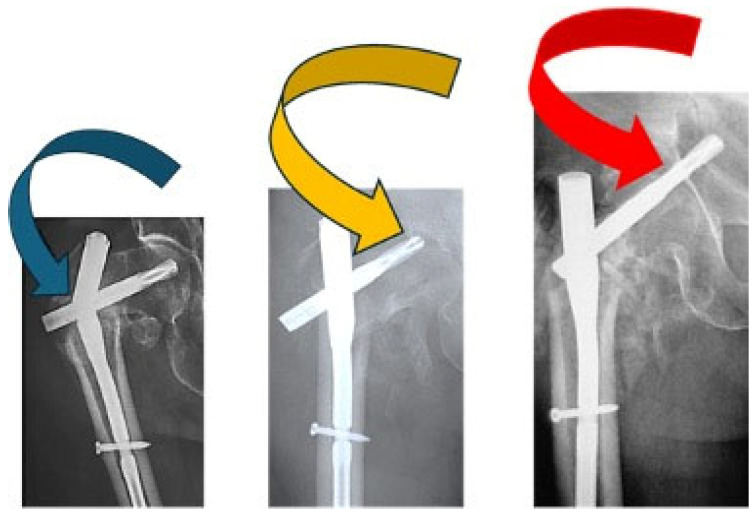
Different failure situations found for proximal femoral nail failure used in intertrochanteric fractures: main nail breakage (blue arrow), helical blade cutout (yellow arrow), and helical blade perforation (red arrow). Adapted from [36].

In Figure 1, different failure situations of the Gamma Nail are presented. Regarding the complications that arise when treating patients with Gamma Nails, the cases associated with osteoporosis or other forms of poor bone quality have an increased chance of implant failure. The implant might loosen or fail if it can not properly stick to the damaged bone. Loosening can also be caused by other factors, such as improper surgical technique, insufficient bone remodeling, infection, implant biocompatibility, and bearing too much weight before the bone has completely healed. Mechanical failure and the need for revision surgery might result from implant corrosion, which slowly degrades the implant components and function [31]. Also, the implant’s components can trigger allergic or inflammatory responses in certain people, which might cause the implant to fail [32]. Gamma Nail failures are rare complications, with incidences of 0.2–5.7% [33,34]. In a series of 2500 Gamma Nail fixations, only 4 (0.16%) Nails eventually broke [16]. The most frequent complications associated with fractures treated with Gamma Nails are the cut out of the cephalic screw (7.1%), diaphyseal fractures (3.1%), and fracture collapses of more than 2 cm (1.5%) [35]. 

For Gamma Nail manufacture, titanium alloy type Ti6Al4V is usually used because it possesses the desired properties for orthopedic implants, like excellent biocompatibility, good corrosion resistance, and high mechanical properties.

Regarding the complications that arise when treating patients with Gamma Nails, it is important to determine the cause in order to be able to avoid them in the future as much as possible. Failure of the nails is most often associated with nonunion (in cases of high energy trauma, associated osteoporosis, and fractures in metastatic bones) and continued weight bearing. Also, the failure of Gamma Nails may appear as a result of implant processing and manufacturing errors or due to technical errors such as a wrong placement of the set screw preventing sliding or inadequate fracture reduction [16]. Different types of tests were conducted using a stereomicroscope, an optical microscope, and scanning electron microscopy in order to reach a conclusion (Figure 1).

## 4. Case Report 

### 4.1. Clinical Case History of the Failed Gamma Nail

**Figure 2:**
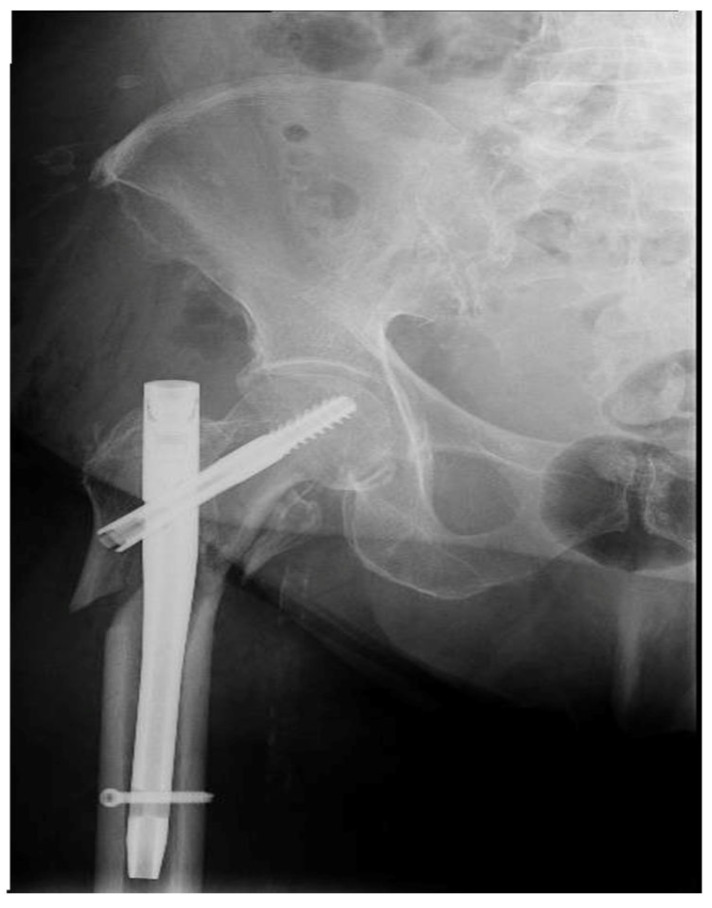
Post surgery X-ray showing the fracture and the position of the implant within the bone.

The reported case presents a 69-year-old woman who suffered a polytrauma as a result of a road accident. The patient suffered a right trochanter femoral fracture that was surgically treated with a Gamma 3 Locking Nail System 11 × 180/125°, lag screw, distal locking screw of 3.5 mm, and Gamma 3 End Cap made of titanium. After the surgical intervention, an X-ray was performed which is shown in (Figure 2).

**Figure 3:**
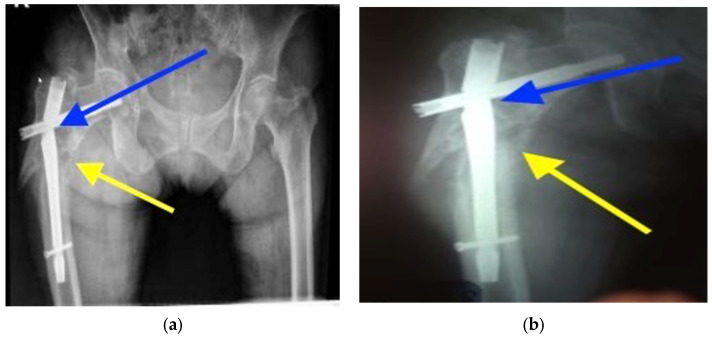
(**a**) Radiographic aspect of the damaged implant (blue arrow) and of the nonunion (yellow arrow). (**b**) Radiographic aspect of the damaged implant (blue arrow) and of the nonunion (yellow arrow).

Six months after the surgery, the patient presented with pain and functional limitation of the right hip. An X-ray, which is shown in Figure 3, was performed, which highlighted nonunion at the fracture level and damage to the implant (Figure 3a,b).

After the X-ray, the removal of the implant was decided, and a surgical correction was performed by changing the implant and stabilizing the fracture with a long DCS system.

**Figure 4:**
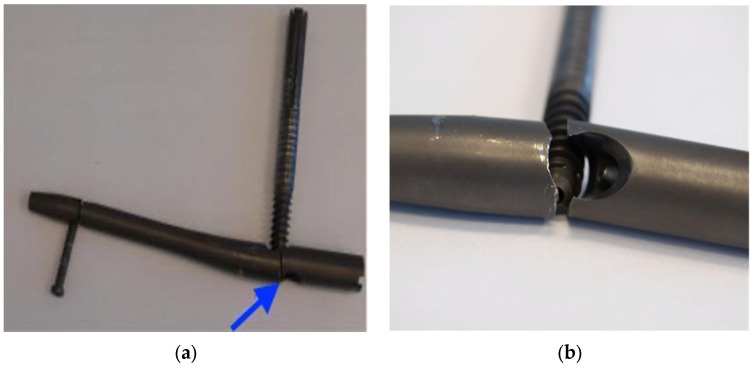
The removed implant: the blue arrow indicates the failure point (**a**). Macroscopic examination after removal (**b**), showing a close-up of the failure point.

After the removal of the broken Gamma Nail, the implant was subjected to some experimental analyses. The broken implant is highlighted in (Figure 4). 

### 4.2. Materials and Method Used for the Analysis of the Failed Gamma Nail

The implant was subjected to analyses using a stereomicroscope, an optical microscope, and scanning electron microscopy in order to study the failure’s cause and mechanism.

The results that were expected to be obtained after the experimental determinations are shown in (Table 3).

In order to highlight the need for using these analysis techniques, every method is briefly explained below.

For the macroscopic analyses of the removed implants, macrophotography and stereomicrosopy were used. The macrophotography was performed using a Canon DSLR with a camera lens macro of 90 mm F 2.8 (Canon Inc., Tokyo, Japan). The stereomicroscopy was performed by using a compact Olympus SZX7 device (Olympus Corporation, Tokyo, Japan). A stereomicroscope is a device utilized for the observation in relief of opaque, translucid, and transparent objects in transmitted light. It is ideal for tridimensional observation [37]. 

Optic microscopy is the most used analysis technique of materials’ microstructure, highlighting morphologic aspects to a resolution between 10 and 2500×, equivalent to dimensions of 0.1–1000 µm. By examining a properly prepared material, at magnifications from about 100× to several thousand times, the phases that make up the material, the grains, the distribution of the phases and grains, their nature, and the size of the crystals can be observed. Because the metallic implants are opaque, the best optical microscopes for examining orthopedic implants are metallographic microscopes, because the examined object is analyzed in reflected light. An Olympus BX51 optic microscope (Olympus Corporation, Tokyo, Japan) was used for these determinations.

Scanning electron microscopy is best used for the analysis of broken implant surfaces. These microscopes have the ability to obtain enlarged images of the investigated probes using an electron beam, thus allowing the examination of very small objects. Also, the possibility to choose which level of the surface of the investigated sample remains in focus from different heights, along with the option of finding the chemical composition of the surfaces, represents another important advantage. A Philips model ESEM XL 30 TMP scanning electron microscope equipped with an EDAX energy spectrometer (Koninklijke Philips N.V, Amsterdam, The Netherlands) was used for these determinations. 

When taking the metallographic samples, the optimal choice for the sampling place and the cutting method was taken into account. For better highlighting of the structure of the implant and of the possible structure variations, the probes were pre-elevated from the immediate vicinity of the broken surfaces. For the optic microscopy, the probes were pre-elevated from the areas that were not affected by the failure. The samples were cut using a Buehler IsoMet4000 device (Buehler, IL, USA) with a special abrasive disc for titanium alloy, and their inclusion parameters are shown in (Table 4). 

The purpose of polishing was to obtain a perfectly flat surface, without scratches, and with a high degree of gloss, and it was performed using a Buehler Beta & Vector Grinder–Polisher (Buehler, IL, USA). The polishing was performed using the parameters shown in Table 5 with metallographic hydrophore paper CarbiMet2 under a continuous stream of water. At the end of the procedure, the sample was washed under running water to remove the traces of the abrasive metal dust and afterwards dried by wiping.

Smoothing aimed to obtain a flat surface with a mirror shine. Smoothing was checked under a microscope at 100× magnification. Non-metallic inclusions, cracks, or preparation defects were observed on the sample. In order to obtain a uniformly smoothed surface, the sample was rotated continuously and in the opposite direction to the rotation of the disc. Otherwise, due to hard constituents, non-smoothed areas may appear as shadows. The smoothed sample with a mirror shine was washed under water, degreased with alcohol, and dried in hot air (Table 5).

**Figure 5:**
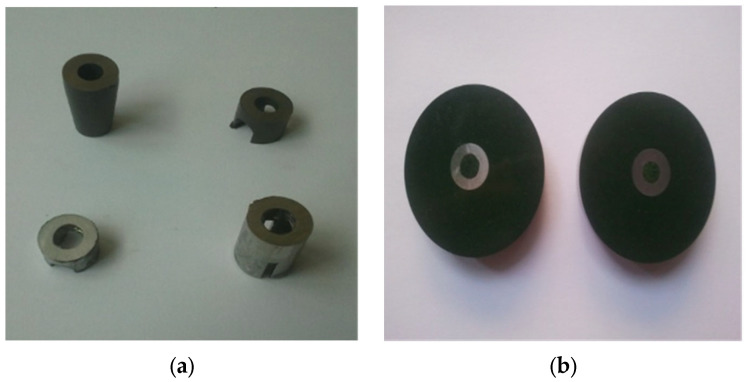
The samples before processing (**a**), and after embedding, polishing, smoothing, and chemical treatment (**b**).

Unlike polishing, which is based on a surface abrasion process, through mechanical smoothing the asperities are leveled by the flow of the material. The surface of the probe was strongly deformed in cold, with the formation of a thin amorphous layer called a Belby layer. This layer, which distorts or obscures the real structure of the probe, is largely removed by metallographic treatment, which aims to highlight the structural constituents. The smoothing parameters of the experimental probes are shown in Table 6, and the probes before and after processing are presented in Figure 4. The experimental titanium alloy samples were chemically treated with a solution containing 100 mL HF and 90 mL of distilled water. Immersion in the solution was for 40 s, and after that the sample was washed in water and dried in a stream of hot air (Table 6), (Figure 5a,b).

### 4.3. Experimental Results Obtained by Microscopical Techniques Regarding the Analysis of the Failed Gamma Nail

#### 4.3.1. Stereomicroscopic Analysis (Figure 6a–d)

**Figure 6:**
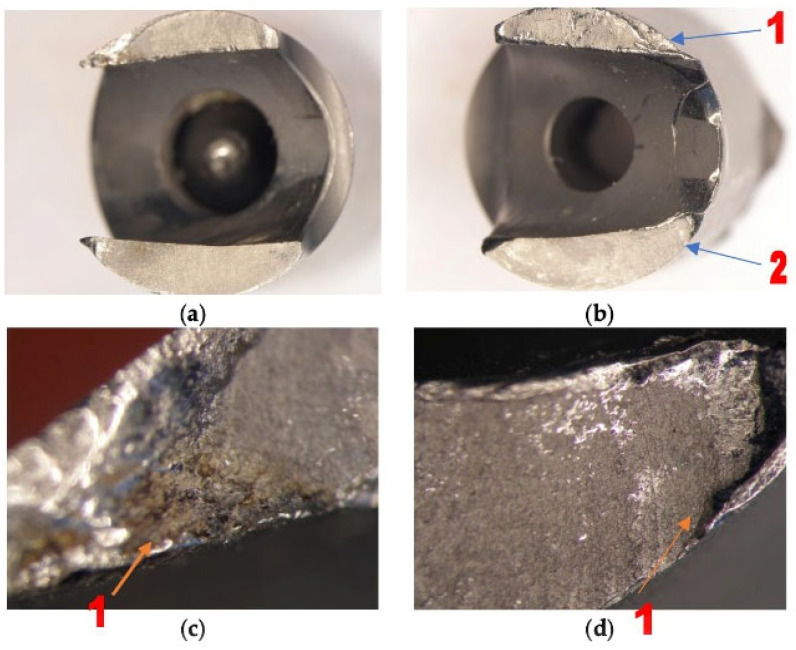
(**a**–**d**) Images obtained by stereomicroscopy of the failure area.

After the stereomicroscopic analysis of the failure surface of the implant, it was observed that there were broken areas, as shown in Figure 6. The component shows gradual breaking due to low and constant energy, with the presence of propagation lines, and then a final, sudden break. The intergranular rupture has a matte aspect and forms three zones: inital, of propagation, and a sudden, final rupture which took place in a short period of time (Figure 6a). It can also be observed that the rupture with intergranular aspects was covered by a thin layer of oxides in Figure 6c(1). In Figure 6b, the fatigue break characteristics, which took place in a short period of time, are highlighted. The failure is mixed: both ductile (1) and brittle (2). The detailed image of the area highlights the cup–cone break type (Figure 6d(1)).

#### 4.3.2. Optical Microscopy Analysis

**Figure 7:**
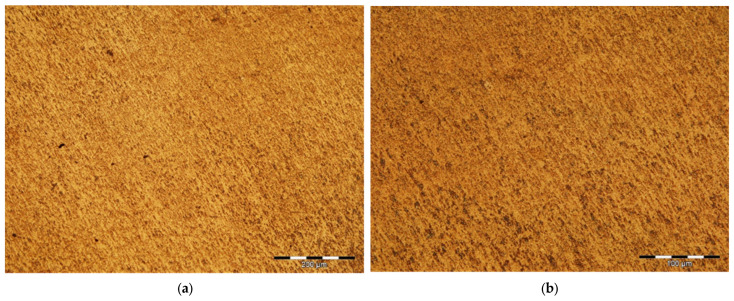

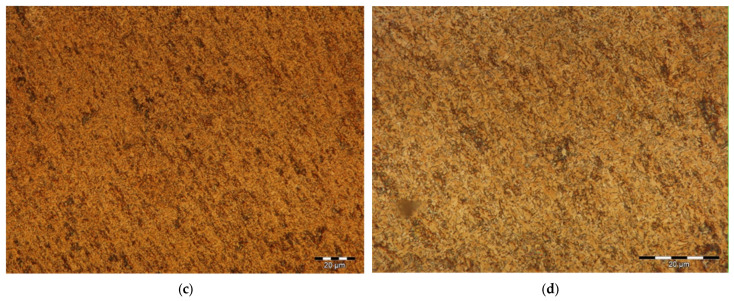
Optical microscopy images related to the microstructural features of the metallic implant material, Ti6Al4V alloy: (**a**) enhancement 100×; (**b**) enhancement 200×; (**c**) enhancement 500×; and (**d**) enhancement 1000×.

The images provided by the optical microscope, presented in (Figure 7), highlight a classic biphasic α + β structure, obtained most probably after a heat treatment for tempering. The elongated appearance of the grains is due to the plastic deformation operations to which the material was subjected (Figure 7a,d).

#### 4.3.3. Scanning Electron Microscopy Analysis

**Figure 8:**
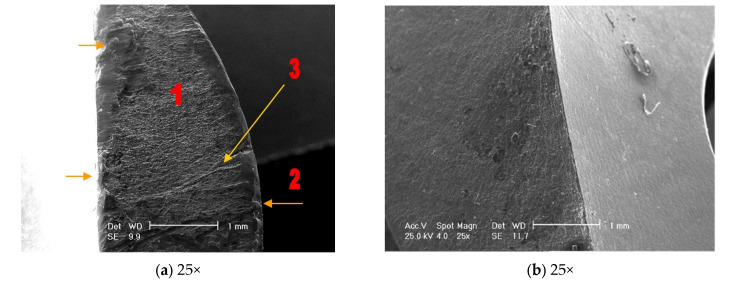

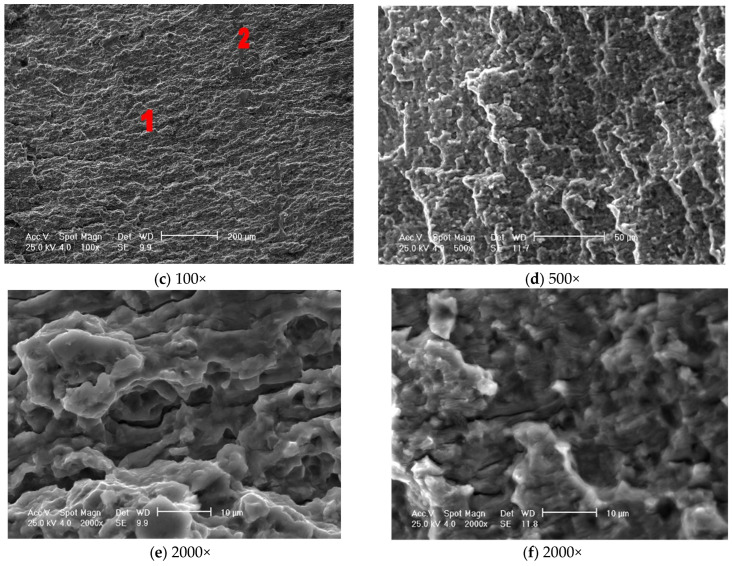
Scanning electron microscopy analyses did not highlight structural inhomogeneities in the rupture area.

Figure 8a shows the intergranular break area, with a matte aspect, covered by a thin layer of oxides (1), which highlight the cup–cone appearance of the rupture (2) as well as the propagation lines of the rupture (3). In image b, the intergranular rupture areas with the presence of small voids in the oxides-covered surface of the sample can be seen. The mixed character of the rupture at 100× magnification can be seen by the presence of brittle (1) and ductile (2) fractures in image c.

Image 8d represents a fracture image magnified by 500×, highlighting relatively equal cups (approximately 10 µm of ductile breakage), as well as relatively parallel rupture zones. In image 8e, the ductile rupture, characterized by the presence of cone–cup type elements, is highlighted. In image 8f, the rupture area at 2000× magnification highlights relatively equal cups (ductile rupture) as well as rupture propagation cracks, both parallel to and in the depth of the sample (Figure 8a–f).

**Figure 9:**
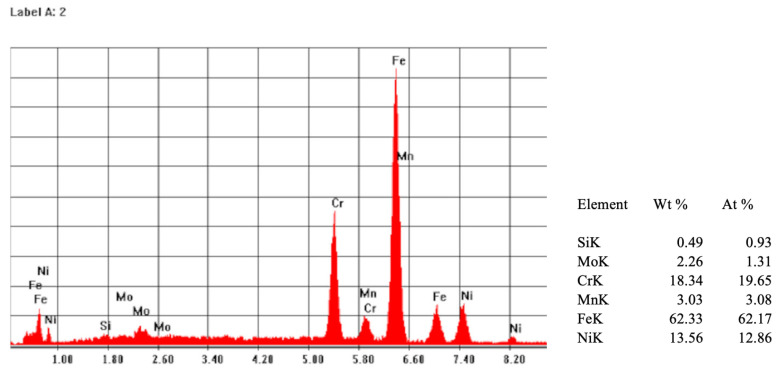
EDS spectrum (**left**) and the results of the compositional analysis of the Gamma Nail’s surface (**right**).

Following the quantitative and qualitative analyses carried out in the failure area and the analysis of the X-ray emission spectrum, the material from which the Gamma Nail was made was evaluated. It was observed that the major components are titanium, aluminium, and vanadium, according to the titanium-based alloys Ti6Al4V highlighted in (Figure 9). 

## 5. Conclusions

The causes that lead to the failure of Gamma Nail implants are different from case to case. The failure of metallic implants, including Gamma Nails, can occur due to stress concentration around a hole made for a screw, which becomes a weak area of the implant, as presented in our reported case. The stress concentration in that weak area can lead to crack initiation, resulting in a fatigue fracture [38]. Also, different microstructural evaluations regarding orthopedic implant holes determined that there is significant plastic deformation in this region, accompanied by the presence of the crevice corrosion phenomenon—pitting corrosion as a result of screw squeeze stress [9].

Another major cause of implant fracture is the failure to comply with the biomechanical criteria between the bone and implant. Regarding implant manufacturing and material defects, there are situations in which the implant was not designed properly or was insufficiently tested before usage. Also, technical errors during surgical intervention can lead to scratches and cracks that can favor subsequent breakage.

Respecting as much as possible the Young’s modulus of elasticity can lead to adequate stiffness matching of orthopedic implants to that of the adjacent bone. Inadequate matching, in which the implant can be significantly stiffer, can lead to implant failure, bone resorption, and stress shielding [39]. Hopefully, the use of pre-clinical experimental tests can lead to personalized treatment, in which more compatible implants will offer better results [40,41].

Also, a discussion topic regarding implant failures consisted of making the implants more rigid and stronger in order to have a better resistance to the forces applied to them. Although it seemed like a good idea at first, it is known that excessively rigid implants can lead to inadequate interfragmentary movement at the fracture or osteotomy site, eventually causing unfavorable healing, remodeling, and stress shielding. Effective bone healing requires controlled interfragmentary movement that takes into account the human bone characteristics [42].

Microscopical tools are very helpful in investigating the failure type and causes. Considering the high morbidity of the trochanteric fractures and of the subsequent implant change, it is very important to find the failure’s cause in order to avoid these situations as much as possible in the future.

After the stereomicroscopy, optical microscopy, and scanning electron microscopy analyses were conducted, imperfections in the preparation and processing of the implant material as causes of the failure were eliminated. The lack of inclusions in the rupture area and of potential structural defects represent proof that the material from which the implant was made was not the cause of its rupture. The failure area is located at the proximal locking system, but there were no signs of excessive friction of the lag screw and no scratches on the surface of the implant that could have been made at the time of insertion into the human body.

Based on our study’s analysis and literature review, we have identified that many incidences of failed Gamma Nails are due to a failure at the lag screw level. This could occur due to stress forces in a high-cycle environment and by errors in using the guiding instruments. In our opinion, the adequate use of the surgical instruments is very important. Even if the forces exerted at this level are not critical after the implantation procedure, after a high number of cycles at a lower stress level, the mechanical resistance of the Gamma Nail is not the same and failure could occur.

Our experimental analyses based on microscopical techniques revealed that the Gamma Nail did comply with regulations from the implant materials point of view, i.e., the chemical composition and microstructure were compliant, and that the implant failed in a ductile mode due to the mechanical overloading caused by surgical errors in fracture reduction and implantation.

Ductile and brittle failure modes are two types of structural collapse that can occur under different loading conditions. Ductile failure involves large deformations and energy dissipation before fracture, while brittle failure involves sudden and catastrophic cracking. Ductile failure is also known as plastic collapse and is the failure mode that occurs when a material is loaded beyond its tensile strength [43]. The stability of the fracture determines the fatigue and stress applied on the Gamma Nail. The implants fail at a lower load in unstable fractures than in stable fractures because the stress on the implant increases with decreasing stability. Therefore, the mechanical support of a fracture is crucial for the fatigue resistance of an implant [44].

In order to be able to avoid this type of complication (that is associated with an increased risk of morbidity due to the advanced ages and comorbidities of these patients), it is recommended that fractures should be very well reduced and that implants should be properly implanted within the bone, so that the tension forces exerted are within the limits supported by the implant and the bone.

## Figures and Tables

**Table 3:** The results that were expected to be obtained after the experimental determinations, depending on the research method used.

Method	Estimated Results
Patient medical data	Patient clinic examination, history, and bone structure information.
Radiologic images study	Visualization of the bone and implant.
EDS microscopy	Determination of the chemical composition and of the metallic material.
Macroscopic analyses by macrophotography and stereomicroscopy	Identification of the failure mechanism. Implant surface failure observation.
Optical microscopy	Identification of microstructural characteristics of the alloys and of the potential structural defects.
Scanning electron microscopy	Identification of the failure mechanisms of the implant. Surface failure observation and considerations about the type of failure. Identification of eventual structural defects in the failure area.

**Table 4:** Inclusion parameters of the experimental samples.

Inclusion Parameters	
Heating time = 8 min	Temperature = 180 °C (350 °F)
Cooling time = 13 min	Phenolic resin
Pressure = 4 atm	Shell size = 40 mm

**Table 5:** Polishing parameters of the experimental samples.

	Polishing 1	Polishing 2	Polishing 3	Polishing 4	Polishing 5	Polishing 6
Time [min]	3	3	3	3	3	3
The force of application [Lb/N]	6	6	6	6	6	6
P [particles/inch] of the Carbi Met paper	400	600	800	1000	1200	2500

**Table 6:** Smoothing parameters of the experimental probes.

	Smoothing 1	Smoothing 2	Smoothing 3
Time [min]	10	10	10
Application force [LB/N]	6	6	6
Material	Tex Met Felt	Tex Met Felt	Tex Met Felt
Smoothing agent	Topol 1*	Topol 2**	Topol 3***

(Topol 1*—emulsion contains alumina particles of 1 µm; Topol 2**—emulsion contains alumina particles of 0.7; and Topol 3***—emulsion contains alumina particles of 0.25).

## Data Availability

All data generated or analyzed during this study are included in this published article.
